# Herpes Simplex Viruses Whose Replication Can Be Deliberately Controlled as Candidate Vaccines

**DOI:** 10.3390/vaccines8020230

**Published:** 2020-05-18

**Authors:** Richard Voellmy, David C Bloom, Nuria Vilaboa

**Affiliations:** 1 HSF Pharmaceuticals SA, 1814 La Tour-de-Peilz, Switzerland; 2 Department of Molecular Genetics and Microbiology, University of Florida College of Medicine, Gainesville, FL 32610-0266, USA; dbloom@ufl.edu; 3 Hospital Universitario La Paz-IdiPAZ, 28046 Madrid, Spain; nuria.vilaboa@salud.madrid.org; 4 CIBER de Bioingenieria, Biomateriales y Nanomedicina, CIBER-BBN, 28046 Madrid, Spain

**Keywords:** herpesvirus, HSV-1, candidate vaccine, vaccine vector, live vaccine, replication-competent controlled

## Abstract

Over the last few years, we have been evaluating a novel paradigm for immunization using viruses or virus-based vectors. Safety is provided not by attenuation or inactivation of vaccine viruses, but by the introduction into the viral genomes of genetic mechanisms that allow for stringent, deliberate spatial and temporal control of virus replication. The resulting replication-competent controlled viruses (RCCVs) can be activated to undergo one or, if desired, several rounds of efficient replication at the inoculation site, but are nonreplicating in the absence of activation. Extrapolating from observations that attenuated replicating viruses are better immunogens than replication-defective or inactivated viruses, it was hypothesized that RCCVs that replicate with wild-type-like efficiency when activated will be even better immunogens. The vigorous replication of the RCCVs should also render heterologous antigens expressed from them highly immunogenic. RCCVs for administration to skin sites or mucosal membranes were constructed using a virulent wild-type HSV-1 strain as the backbone. The recombinants are activated by a localized heat treatment to the inoculation site in the presence of a small-molecule regulator (SMR). Derivatives expressing influenza virus antigens were also prepared. Immunization/challenge experiments in mouse models revealed that the activated RCCVs induced far better protective immune responses against themselves as well as against the heterologous antigens they express than unactivated RCCVs or a replication-defective HSV-1 strain. Neutralizing antibody and proliferation responses mirrored these findings. We believe that the data obtained so far warrant further research to explore the possibility of developing effective RCCV-based vaccines directed to herpetic diseases and/or diseases caused by other pathogens.

## 1. Introduction

For a number of important diseases there exist only vaccines that are at best moderately effective and/or require frequent updating and re-vaccination. For some of diseases, no vaccines are available today, and this is not due to insufficient efforts by the academic and industrial communities. Herpetic diseases mediated by HSV-1 and HSV-2 belong to the latter category of diseases for which no vaccine has been successfully developed. This state of affairs should encourage searches for approaches that differ qualitatively from what has been explored before.

## 2. The Concept

Immunization with an enriched or pure protein of a pathogen tends to favor a humoral immune response over a cell-mediated immune response, i.e., a Th2-biased response instead of a more balanced Th1 and Th2 response. To mitigate this deficiency, vaccines containing split, subunit, or recombinant antigens are typically adjuvanted, which introduces the possibility of adjuvant-associated health problems that may surface over the life of a vaccinated individual [1].

Historically, inactivated and attenuated pathogens were developed as vaccines. Not surprisingly, debates ensued regarding the relative efficacies of vaccines containing inactivated pathogens and vaccines containing attenuated pathogens. The best-remembered debate of this kind concerned the inactivated poliomyelitis vaccine of Salk and the live attenuated vaccine of Sabin [2,3]. The history of vaccination against measles provides another early case for the apparent superiority of a live attenuated vaccine over an inactivated vaccine. A formalin-inactivated measles virus vaccine licensed in the USA was withdrawn in 1963 in part because it only induced short-lived immunity, and in part because certain vaccinated individuals experienced an atypical measles syndrome upon subsequent natural exposure to the measles virus [4,5]. The inactivated virus vaccine was subsequently replaced by a live vaccine derived from the attenuated Edmonston B strain (isolated in 1954), which remains in use today. The current prevailing view appears to be that live attenuated vaccines generally induce a more balanced immune response and provide better and longer-lasting protection than inactivated virus vaccines. Successful live attenuated vaccines include vaccines against measles, mumps, rubella, smallpox, and yellow fever [6].

The key difference between inactivated and live attenuated viral vaccines is that the live attenuated vaccines have retained some capacity for replication. However, the two types of vaccines also differ in other respects. The process of inactivation may alter the antigens presented by an inactivated vaccine, whereas the surface of live attenuated vaccines is unadulterated. Furthermore, inactivated and live attenuated vaccines against a particular pathogen may be administered by different routes, as is the case for the poliomyelitis vaccines. Hence, the superiority of live attenuated vaccines over inactivated vaccines cannot be unambiguously ascribed to their residual ability to replicate. However, a number of studies have addressed the issue more directly, comparing immune responses to live attenuated viruses that retained some ability to replicate and corresponding replication-deficient viruses or to heterologous antigens expressed by them [7,8,9,10]. These studies confirmed the expectation that live attenuated viruses induce more potent and/or more balanced immune responses (to the viruses themselves or to expressed heterologous antigens) than the non-replicating comparison viruses.

We hypothesized that if a replication-attenuated virus produces a more potent and/or more balanced immune response than a non-replicating virus, this immune response might be further enhanced by immunization with a virus that is capable of replicating, for a limited time, with a wild-type-like efficiency [11]. This hypothesis was based on the rational expectation that such a virus would induce more potent inflammatory and innate immune responses and, consequently, a stronger adaptive immune response than an attenuated or replication-deficient virus, resulting in an enhanced preventative or therapeutic efficacy. It seemed plausible that inflammatory and innate immune responses would be triggered most effectively if replication of the virus was concentrated in a particular region, i.e., the administration site and the tissue closely adjacent to or surrounding the administration site, rather than being allowed to occur in a disseminated fashion. To test this hypothesis, a vaccine virus, referred to herein as a replication-competent controlled virus (RCCV), had to be constructed whose replication is under stringent, deliberate temporal and spatial control. To achieve this, one or more replication-essential genes of the virus of choice needed to be subjected to a genetic control mechanism that essentially functions as an on–off switch as well as allows for local actuation.

Alphaherpesviruses such as HSV-1 or HSV-2 appeared to be well-suited to serve as backbones for the construction of RCCVs for a number of reasons. They are double-stranded DNA viruses that employ the cellular transcription machinery for expression of their genes [12,13,14]. Hence, well-tested gene switches that function in mammalian cells may be adapted for the control of one or more replication-essential viral genes. The viral DNA remains episomal. Therefore, the complication of deleterious insertions of viral DNA into the host-cell genome may be avoided. The viruses replicate well in a variety of cell types including, in particular, epithelial cells, as well as lysing cells efficiently. The viral genomes are tolerant of sizeable insertions, allowing for the introduction of control elements and passenger genes essentially without affecting efficiency of replication. The viruses typically do not cause lethality, and additional safety is provided by antiviral drugs that are readily available. On balance, the fact that alphaherpesviruses, in particular HSV-1, are prevalent in the human population may be considered to be an advantage. Finally, by their nature, RCCVs based on alphaherpesviruses will express all antigens of these viruses, which should be an important advantage when uses as anti-herpetic vaccines are contemplated. The alternative of employing another virus as a vector for one or even several herpesvirus antigens would appear to be less desirable. The fact that alphaherpesviruses are capable of latent infection may be considered to represent but a minor risk; if a genetic mechanism can be adapted to effectively control lytic replication, this mechanism should also be capable of preventing reactivation from latency. All RCCVs that we have constructed to date are based on a wild-type HSV-1 strain. Hence, the following discussion focuses on HSV-1, and viral genes identified relate to genes present in the HSV-1 genome.

### 2.1. A Priori Concerns Regarding Possible Obstacles to the Successful Development of RCCVs

At the outset, there were several reasons for doubting that the above-described vaccination strategy could be successfully developed. One such concern was that it might not be possible to build RCCVs whose replication can be controlled with the appropriate stringency. The choice of suitable gene switches appeared to be rather limited. Subjecting replication of an RCCV to a drug-activated mechanism was considered to be unlikely to produce the desired sharp regulation. Before the concentration of a systemically administered drug had fallen below an effective level, the RCCV might have undergone multiple rounds of replication. Additionally, the rate of clearance of a drug may vary sufficiently between individuals to render the elaboration of a standard vaccination protocol difficult or unfeasible. A somewhat better but probably still inadequate control could be achieved by topical administration of the drug in a timed-release formulation. Only a physical cue or stimulus whose application can be precisely timed and confined to the RCCV administration site would provide for a sufficiently stringent temporal and spatial regulation of RCCV replication, provided that the response to the trigger subsides before infectious progeny of the RCCV are generated.

A physical cue or stimulus that is applied to the virus administration site might impair the activity of cells of the innate immune system, including resident antigen-presenting cells, dampening any immune response that might be induced by the RCCV.

Another question that could not be answered a priori was whether limited replication of an RCCV confined to the site of its administration could in fact produce a significant enhancement of the immune response to the virus. No information pertinent to this question was available at the time. Attenuated replicating vaccines (or candidate vaccines), including single-cycle vaccines, replicate systemically.

Regarding the quality and potency of inflammatory and immune responses induced by an activated (i.e., replicating) RCCV, it could not be predicted with any confidence how much better they would be compared to responses induced by more conventional vaccines. On the one hand, efficient but limited replication of an RCVV that is concentrated in the administration region will result in localized lysis of many infected or superinfected cells and spillage of virus progeny and cell contents. This will result in very high local levels of PAMPs and DAMPs that can be expected to prompt a strong inflammatory response and stimulation of the innate immune system. Primarily infected apoptotic antigen-presenting cells such as dendritic cells may efficiently cross-present viral antigens to uninfected dendritic cells [15]. Presentation of viral antigens by primarily infected nonprofessional antigen-presenting cells (e.g., epithelial cells if virus is administered to a skin site) may play a lesser role. Efficient expression of viral proteins such as ICP47, US3, and vhs can be expected to inhibit MHC-class-I antigen presentation [16,17,18,19]. However, bystander cells may be secondarily infected by the now-abundant RCCVs. By the time secondary infection can take place, the locally activated mechanism that supported RCCV replication during the primary infection cycle will have returned to an inactive state. Hence, secondarily infecting RCCVs will not replicate and not express immunomodulatory proteins at highly levels. Depending on which viral gene is deliberately regulated, some or most immunomodulatory viral genes will not be expressed at all. Secondarily infected professional antigen-presenting cells should therefore be capable of surviving and traveling to a lymph node for efficient B- and T-cell activation [20].

On the other hand, viral immunomodulatory proteins that will be abundantly expressed in primarily infected cells may severely inhibit cytokine and interferon production (and signaling), thereby dampening the (initial) inflammatory and immune responses. Pattern recognition receptor and/or adapter function is inhibited by viral proteins UL37, ICP0, UL36USP, US3, and ICP34.5; interferon-regulatory factors (IRF) are interfered with by US3, US11, ICP34.5, VP24, ICP27, and ICP0; and NF-kB functionality is inhibited by UL36USP, US3, and ICP0. Furthermore, JAK/STAT signaling is blunted by the action of viral proteins VR3, UL13, UL41, and ICP27 [21] (see also References [22,23]).

Another concern was that the locally elevated concentrations of PAMPs and DAMPs resulting from efficient localized virus replication might trigger an excessive local inflammatory response/release of pro-inflammatory cytokines. 

Results that have been obtained to date (discussed below) indicate that RCCVs can be constructed whose replication is under sufficiently stringent deliberate control to provide the requisite safety as well as support our hypothesis that such recombinant viruses are capable of inducing superior immune responses without any apparent adverse effects. We submit that these results lay the foundation for the further development of RCCVs as candidate vaccines or vaccine vectors.

### 2.2. Note on Additional Expected Advantages of RCCVs

RCCV-based vaccination may be accompanied by several additional important advantages:

There is a recognized risk of run-away replication associated with vaccinating immunocompromised individuals with attenuated replicating viruses [24]. This danger should not exist for RCCVs, as their replication is under deliberate exogenous control.

For the same reason, RCCVs should be resistant to reactivation from quiescence in infected nerve cells.

RCCVs are activated in a chosen administration site. They should not replicate in a disseminated fashion. Hence, they are not expected to exhibit residual neurotoxicity, which has been observed even with single-cycle vaccines [25]. 

As activated RCCVs will only replicate locally and for a limited time (typically the time required for the completion of a round of replication), there will be little opportunity for recombination with another virus. In the absence of activation, RCCVs are expected to be only present at low copy numbers in infected cells.

## 3. Development of RCCVs

### 3.1. RCCVs Controlled by Heat

The host mechanism that controls the expression of heat shock genes appeared to be ideally suited for controlling RCCV replication. The inducible transcription of heat shock genes, a group that includes the classical HSP genes as well as many additional genes, is under the control of heat shock transcription factor 1 (HSF1) [26]. This ubiquitously expressed transcription factor is normally present in cells as a dynamic heterocomplex comprising HSP90 and other proteins. In this form, HSF1 is transcriptionally inactive. Activation results in a rapid release of HSF1 from the heterocomplex, whereupon the factor homo-oligomerizes and undergoes phosphorylation and other modifications, thereby acquiring the ability to transactivate heat shock genes. Heat is by far the most effective trigger of HSF1 activation. In this context “heat” refers to an exposure of cells to a temperature that is several °C above the temperature of their natural environment. Activation of HSF1 by heat is a transient response that subsides in at most a few hours [27,28]. Rapid inactivation of HSF1 occurs even in cells that are continuously exposed to mild heat. The promoters of certain heat shock genes are essentially on–off switches, i.e., they exhibit an exceedingly wide dynamic range. Some of these promoters appear to be exclusively (to the extent that this is known) transactivated by activated HSF1. For example, the promoter of the human *HSP70B* (*HSPA7*) gene has a very low basal activity in cultured cells and a heat-induced activity that is almost 1000-fold higher [26,29,30]. In the skin of human subjects, topical heat treatment was found to increase the activity of the latter promoter several hundred times [31]. Significant activation of the promoter in human cells requires heat treatments at temperatures exceeding 42 °C, i.e., at temperatures that only occur in patients experiencing hyperpyrexia.

Because alphaherpesviruses infect and replicate well in epithelial cells, an HSV-1-based RCCV may be administered preferably to a site in the skin or, possibly, a mucosal tissue. Heat delivery can be readily directed, in particular to skin sites. For example, disposable/recyclable or reusable heating pads may be employed. A pad that contains a supercooled solution of the essentially nontoxic salt sodium thiosulfate pentahydrate has been tested in human subjects [31]. After crystallization is triggered mechanically, the pad heats up rapidly and maintains a surface temperature of about 45 °C for at least 15 min. A 15 min exposure of a skin region to this pad resulted in a strong transcriptional activation of the *HSP70B* gene in this region. Activation of HSF1 is a proportional response to proteotoxic stress. Hence, the degree of activation is a function of heat dose, not temperature alone. Consequently, heating time could be decreased by increasing exposure temperature. In animal experiments employing high-intensity focused ultrasound, activation of the human *HSP70B* promoter could be achieved in discrete tissue regions by a 3 min exposure [32]. Activation of HSP promoters in the skin of experimental animals by mid-IR or near-IR laser irradiation was apparent after exposures in the second- or even sub-second range [33,34,35].

**Figure 1:**
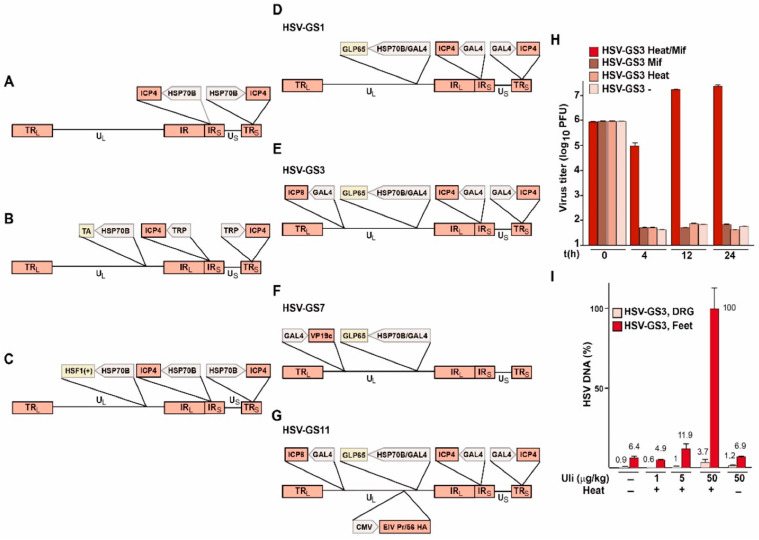
(**A**–**G**) Schematic structures of RCCVs. Transactivators: TA (unspecified transactivator), HSF1+ (constitutively active HSF1 mutant), GLP65 (antiprogestin-activated transactivator) [37,38]; promoters: HSP70B (promoter of the human HSP70B gene), TRP (transactivator-responsive promoter), GAL4 (GLP65-responsive promoter), CMV (cytomegalovirus immediate early promoter); influenza virus gene: EIV PR/56 HA; backbone virus: named genes: ICP4, ICP8 and VP19c, structural elements: U: unique sequences, TR/IR: repeat sequences. (**H**) Single-step growth experiment with HSV-GS3 in human SSC-15 cells. Four basic conditions were tested: (i) heat treatment at 43.5 °C for 30 min in the presence of 10 nM mifepristone (Mif) (activating treatment), (ii) heat treatment alone, (iii) mifepristone exposure alone, and (iv) no treatment. Heat treatment was administered immediately after infection (i.e., immediately after removal of the viral inoculum). At 0, 4, 12, and 24 h post-infection, duplicate dishes were removed, and the cells were scraped into medium for harvesting and subjected to two freeze–thaw cycles. Infectious virus levels were then determined by titrating the lysate of each dish in triplicate on 24 well plates of confluent E5 cells (ICP4-expressing cells) transfected with an ICP8 expression construct. Plaques were visualized after 2 days by staining with crystal violet. (**I**) DNA replication of HSV-GS3 in a mouse footpad model. Adult outbred mice were inoculated on the slightly abraded footpads of their rear feet with 10^5^ PFU of HSV-GS3. The indicated doses of ulipristal (Uli) were administered intraperitoneally at the time of infection. Localized heat treatment at 45 °C for 10 min was performed 3 h after virus administration. Mice were sacrificed 24 h after heat treatment, and DNA was isolated from feet and dorsal root ganglia (DRG) and analyzed by qPCR. Values and standard deviations were normalized relative to the highest value. Panels H and I have been reproduced in slightly altered form from Reference [39]. See Reference [39] for additional experimental detail.

A simple version of an RCCV may be generated by replacing, by homologous recombination, a promoter of a replication-essential gene in a wild-type HSV-1 strain with a human *HSP70B* promoter (Figure 1A). Following cutaneous or subcutaneous administration of the RCCV, an appropriate heat dose would be applied to the administration region. This would result in an activation of HSF1 in infected as well as uninfected cells within the administration region (but not elsewhere in the body of the inoculated subject). Viral genes including the regulated replication-essential gene would be expressed in the infected cells and, hopefully, progeny virus would be produced with an efficiency similar to that of the wild-type virus. Some sensory neurons within the administration region would be quiescently infected. Progeny virus would infect other permissive cells. This secondary infection would take place, at the earliest, half a day after the heat treatment (i.e., after completion of a round of replication in the primarily infected cells), at which time HSF1 would have long since returned to its inactive state. Consequently, RCCVs would not replicate in the secondarily infected cells. 

Conceivably, replication of the latter simple RCCV might not be as efficient as would be desired because transcription of the regulated viral gene would cease shortly after the heat treatment. The resulting concentration profile of the protein product of the gene might be suboptimal or might even be incompatible with viral replication if the regulated gene were a late gene. This potential problem could be avoided or at least mitigated by constructing an RCCV that contains an inserted *HSP70B* promoter-driven gene for a heterologous transactivator, and in which the promoter of the viral gene to be regulated has been replaced with a transactivator-responsive promoter (Figure 1B). To ensure that the transactivator is maintained at an elevated level throughout the viral replication cycle, the gene for the transactivator could be additionally subjected to autoactivation. The feasibility of building a stable regulatory circuit of this type has been demonstrated previously, although the dynamic range of the circuit appeared to be somewhat narrower than that of the *HSP70B* promoter (Reference [36]; Figure 1); the circuit comprised an *HSP70B* promoter-linked gene for a mutated, constitutively active HSF1 and an *HSP70B*-promoter-controlled reporter gene. In cells containing this circuit, the reporter was expressed at a high rate for several days after a single activating heat treatment. Hence, an RCCV could be built by insertion into the genome of a wild-type virus strain of an *HSP70B* promoter-controlled gene for a constitutively active HSF1 and replacement of the promoter of a replication-essential viral gene with an *HSP70B* promoter (Figure 1C).

### 3.2. RCCVs Dually Controlled by Heat and a Drug (Antiprogestin)

While such an RCCV may be adequately controlled under normal circumstances, nobody would dare to propose the development of a vaccine that comprises a fully virulent virus that is singly controlled by an HSP promoter: HSF1 activation occurs in response to proteotoxic stress, which is not only caused by heat but also by intoxication and, possibly, other disturbances of cellular homeostasis. Furthermore, nobody knows how strongly HSF1 is activated in infected nerve cells of a feverish subject. Therefore, a viable candidate vaccine would need to incorporate one or more fail-safe mechanisms. The RCCVs actually built employed small-molecule regulator (SMR)-activated transactivator GLP65 to mediate the expression of one or more replication-essential viral genes. Expression of the transactivator was placed under the control of a promoter assembly containing an *HSP70B* promoter and a transactivator-responsive promoter [36]. In cells infected with these RCCVs, replication can be triggered by a heat treatment of the cells in the presence of an effective concentration of the SMR. Replication is not induced in the absence of either cue. Transactivator GLP65 comprises a GAL4 DNA-binding domain, a truncated ligand-binding domain of a human progesterone receptor, and an activation domain from human NF-kB p65 [37,38]. This transactivator was selected in large part because of the properties of its SMR, which belongs to a narrow class of synthetic antiprogestins. Representatives of this class are mifepristone and ulipristal. Other reasons included the fact that GLP65 is virtually devoid of transactivation activity in the absence of its SMR and, to the best of our knowledge, it is not susceptible to SMR-independent activation. The truncated progesterone receptor ligand-binding domain lacks activation functions AF-1 and AF-2, as well as all sites of which phosphorylation promotes receptor activity [11]. An ideal SMR would be a drug-like molecule that is essentially devoid of toxicity and that is not otherwise employed in human medicine. However, if a transactivator that is activated by such a molecule were employed, development of an RCCV-based vaccine would also involve a full preclinical and clinical investigation of the SMR. More practically, a suitable SMR will have to be an approved drug that is used only sporadically and in single-dose regimens. Mifepristone and ulipristal are approved drugs that are used primarily in emergency contraception. 

The structures of RCCVs HSV-GS1, HSV-GS3, and HSV-GS7 are depicted schematically in Figure 1D–F. The recombinants were derived from virulent wild-type HSV-1 virus strain 17*syn*+ [39,40,41]. All constructs contain, inserted in the UL43/44 intergenic region, an *HSP70B/GAL4* promoter assembly that is functionally linked to a *GLP65* gene. The native promoters of the regulated replication-essential genes were replaced by GAL4-responsive promoters. The regulated genes are the immediate early gene *ICP4* in HSV-GS1 and HSV-GS3, the early gene *ICP8* in HSV-GS3, and the late gene *UL38/VP19c* in HSV-GS7. Note that in HSV-GS3, two replication-essential genes were subjected to regulation, eliminating the possibility that a single mutation could give rise to an unregulated virus. 

Single-cycle growth experiments demonstrated that the RCCVs replicated efficiently in cells that had received an activating heat treatment and were exposed to an antiprogestin [39,40]. Comparison experiments revealed that the replication efficiencies of the activated RCCVs were comparable to that of wild-type virus 17*syn*+. Viral replication was not observed in untreated infected cells or in infected cells that were only heat-treated or only incubated in the presence of an antiprogestin. As an illustration, results from a single-step growth experiment in HSV-GS3-infected cells of a permissive human cell line are represented in Figure 1H (originally reported in Reference [39]). The same stringent regulation of replication was also observed in vivo. Replication only occurred in the presence of an adequate concentration of an antiprogestin and subsequent to a localized heat treatment applied to the virus administration site. Results from a representative experiment are shown in Figure 1I (originally reported in Reference [39]). In this experiment, adult mice were inoculated on their slightly abraded rear footpads with RCCV HSV-GS3 as well as being dosed with ulipristal, as indicated in Figure 1I. Three hours after virus administration, the mice of some groups were subjected to a 45 °C/10 min heat treatment to their rear feet. Twenty-four hours later, all animals were sacrificed, and DNA and RNA were extracted from feet and dorsal root ganglia and analyzed by PCR and RT-qPCR, respectively. Figure 1I represents relative amounts of viral DNA recovered after different treatment regimes.

## 4. Immune Responses to Activated RCCVs

Most of the results discussed under this heading were originally disclosed in References [39,40,41].

### 4.1. Anti-Herpetic Immune Response

**Figure 2:**
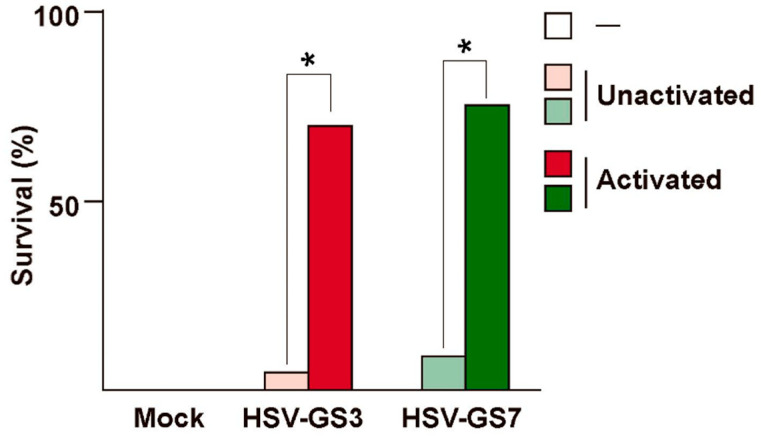
Protective immunity induced by RCCVs HSV-GS3 and HSV-GS7. Mice were inoculated on both rear feet with 50,000 PFU/mouse of either HSV-GS3 or HSV-GS7, or were mock-immunized with saline. For each recombinant, one group of animals was subjected to heat treatment in the presence of ulipristal (activated) and another group did not receive this treatment (unactivated). At 21 days post-inoculation, all mice were challenged with a 20-fold lethal dose of wild-type HSV-1 strain 17*syn*+ applied to both rear feet. The data are presented as percent survival (at 26 days post challenge) for each treatment group (*n* = 20 for each HSV-GS3 or HSV-GS7 group; *n* = 10 for the mock-immunized group; *, *p ≤* 0.05). This figure is based on data reported in Reference [40].

The immune response to activated RCCVs was evaluated in a stringent mouse footpad lethal challenge model [40]. In one such experiment, groups of adult outbred mice were inoculated with RCCVs HSV-GS3 or HSV-GS7 (50,000 PFU) on the footpads of their rear feet. RCCV activation involved intraperitoneal administration of ulipristal at the time of virus inoculation and a heat treatment to the rear feet three hours later. No signs of inflammation or swelling of the feet, or other signs of disease or discomfort were observed in RCCV-inoculated mice. Three weeks after RCCV administration, the mice were challenged with a 20-fold lethal dose of wild-type virus 17*syn*+ administered to the rear feet. All animals developed signs of illness. Seventy-five percent of the mice immunized with activated HSV-GS7 and seventy percent of the mice immunized with activated HSV-GS3 recovered fully (Figure 2). Without activation, survival rates were 10% and 5%, respectively. Complete protection was observed in animals that were immunized twice with activated RCCV (incidentally demonstrating that a significant immune response could be elicited in the presence of pre-existing immunity). The latter findings suggest that the protective response was largely cell-mediated, which is perhaps not a surprise. Regarding correlates of the protective response, activated HSV-GS3 and HSV-GS7 were found to induce considerably higher levels of HSV-1-specific neutralizing antibodies than the unactivated viruses or a replication-defective control virus. Lymphoproliferation assays revealed that the cellular responses to the activated recombinants were substantially stronger than those induced by the unactivated recombinants or the replication-defective control virus. 

Our experiments demonstrated that, via their efficient but spatially and temporally limited replication, RCCVs induced substantially greater protective immune responses than non-replicating vectors. The enhanced activation of the immune system caused by the replicating viruses trumped the viral defenses that work to inhibit inflammatory and innate immune responses. Apparently, even at the elevated levels to which they accumulate during virus replication, the immunomodulatory viral proteins were unable to neutralize the immune-enhancing effect of replication. It may be worth mentioning that our experiments also did not discover any evidence for the efficacy of viral defenses against unactivated, i.e., non-replicating, RCCVs: unactivated HSV-GS7 should express all viral proteins with the exception of capsid protein VP19c, whereas unactivated HSV-GS3 should not express any viral proteins other than the immediate early proteins ICP0 and ICP27. The protective immune responses induced by the two recombinants were comparable. However, it is noted that this finding does not speak to the role of ICP47, which blocks peptide binding to the transporter associated with antigen processing (TAP). Viral protein ICP47 is known to contribute importantly to the viral defense in humans, an effect that is not recapitulated in mouse models [17,18]. ICP47 is not an essential viral protein. RCCVs intended for human use may contain a mutated ICP47 gene encoding a protein that is no longer capable of interacting with the TAP transporter.

Regarding other initial concerns, our findings imply that a single round of spatially confined virus replication is indeed capable of inducing a greatly enhanced protective immune response. Furthermore, even if the RCCV activation procedure had any negative effect on the immune system, this effect was clearly not dominant. 

To summarize, our experiments provided strong evidence that an activated RCCV that undergoes efficient but limited replication is capable of inducing a superior functional immune response. Our findings motivate further studies employing animal models of specific herpetic diseases, e.g., models of ocular or genital herpes. Such studies may also address the important question of whether the enhanced immunity resulting from immunization with an activated RCCV will suffice to prevent or at least substantially mitigate reactivation of wild-type virus from latency.

### 4.2. Immune Response to a Vectored Antigen of Another Pathogen

**Figure 3:**
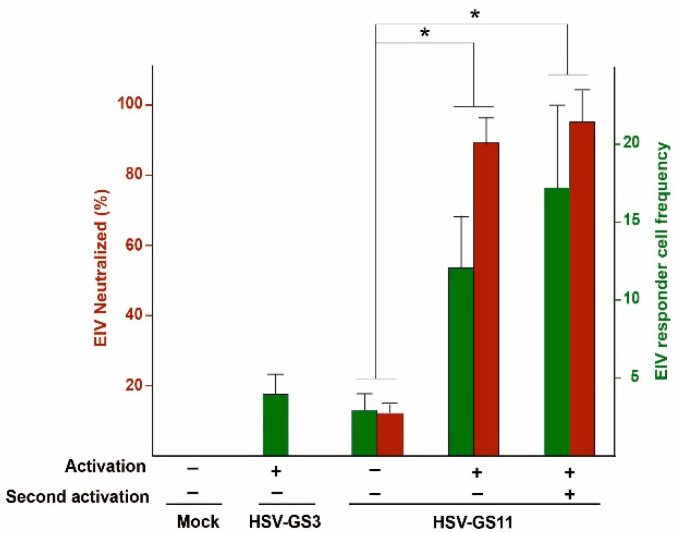
Immunization with activated HSV-GS11 induced a robust immune response against EIV Prague/56 HA. For neutralizing antibody assessments: groups (*n* = 5) of 6 to 8 week old female BALB/c mice were inoculated on both rear footpads with either saline (mock), HSV-GS3 (50,000 PFU), or HSV-GS11 (50,000 PFU). Vector replication was activated in some treatment groups by administration of heat and ulipristal. One treatment group received a second activation treatment that was administered 2 days after the first activation treatment. Twenty-one days post-immunization, mouse serum samples were tested for their ability to neutralize EIV Prague/56. The values are presented as percentages of EIV Prague/56 neutralized. For responder cell frequency assays, immunizations and treatments were as above. Twenty-one days post-immunization, the number of EIV Prague/56 HA-specific lymphocytes was determined using a limiting dilution lymphocyte proliferation assay. The data are presented as the responder cell frequency of each experimental group; *, *p ≤* 0.05). See Reference [40] for the original report of the results shown in this figure.

If the efficient but limited replication of an RCCV results in a substantially enhanced immunity against the type of herpesvirus from which the RCCV was derived when compared to the immunity induced by a non-replicating herpesvirus, one might expect a correspondingly strengthened immune response against a heterologous antigen if it is expressed from a replicating RCCV. Hence, RCCVs should be potent carriers of heterologous antigens. To test this hypothesis, expressible genes for the hemagglutinin (HA) or the nucleoprotein (NP) of equine influenza virus Prague/56 (EIV Prague/56) were introduced into the UL37/38 intergenic region of RCCV HSV-GS3 (see Figure 1G for HSV-GS11, a recombinant expressing HA). Mice were inoculated on their rear feet with the heterologous antigen-carrying recombinants which were either activated (once or twice) or left unactivated. HSV-GS3 served as a control. Three weeks later, neutralizing antibody titers were assessed and responder cell frequencies determined in a lymphoproliferation assay. Results obtained from such an experiment employing recombinant HSV-GS11 are represented in Figure 3 (originally reported in Reference [40] Immunization with activated HSV-GS11 induced a several-fold higher level of EIV-specific neutralizing antibodies than immunization with unactivated HSV-GS11. No neutralizing antibodies were detected in sera from animals immunized with HSV-GS3. Similarly, the level of HA-specific responder cells was much higher in PBMCs from animals immunized with activated HSV-GS11 than in PBMCs from animals immunized with the unactivated vector. For reasons to be explored, a weak response was also induced by HSV-GS3. Hence, efficient but limited vector replication resulted in an impressive enhancement of HA-specific humoral and cellular responses. 

More recent experiments (unpublished) evaluated the functional immune responses to EIV antigens expressed from activated RCCV vectors using a mouse model that had been described previously [42]. In these experiments, mice immunized with activated vectors were challenged intranasally with a lethal dose of EIV Prague/56. Survival of the animals was followed until no more lethal endpoints (>20% weight loss) were reached and animals had fully recovered. Nearly all animals immunized with an activated vector expressing the EIV HA or NP survived the challenge (100% of HA-immunized animals and 90% of NP-immunized animals). The weight loss curves suggested that the HA but not the NP induced sterile immunity. Evidence was also obtained for heterosubtypic protection. In these experiments, mice were immunized with activated RCCVs expressing the HA or the NP of EIV Kentucky/94 (H3N8) and were challenged with a lethal dose of EIV Prague/56 (H7N7).

Based on results obtained to date, we submit that RCCVs are powerful vectors of heterologous antigens. Regarding influenza, RCCVs expressing surface or internal influenza virus antigens may be considered as candidates for effective anti-influenza vaccines that may be well suited for the vaccination of the immunocompromised and senior populations. Whether such vaccines will also be beneficial to the general population may depend in part on the breath of protection they are capable of providing, i.e., whether they could be employable as non-seasonal influenza vaccines. Current work is aimed at defining the boundaries of the cross-protective activities of influenza virus antigen-expressing RCCVs. 

## 5. Further Thoughts—Conclusions

At this time, heat appears to be the only physical cue that can be employed for the stringent spatial and temporal control of RCCV replication. This is due to two properties of the heat shock system, i.e., the existence of HSP promoters that essentially behave as on–off switches and the transience of the activated state of HSF1, the transcription factor that transactivates HSP promoters.

While it may be possible to build/employ other safeguards against inadvertent stress activation, we chose to co-control RCCV activation using a drug-mediated mechanism. One or more replication-essential viral genes are placed under the control of an SMR-activated transactivator that is expressed from a gene that is driven by an HSP promoter. The chosen transactivator comprises a GAL4 DNA-binding domain and a truncated progesterone receptor ligand-binding domain, and is activated by a narrow class of antiprogestins that include mifepristone and ulipristal. Replication-essential genes to be controlled by this transactivator have their native promoters replaced by minimal GAL4-responsive promoters, i.e., promoters that are bound by the GAL4 DNA-binding domain of the transactivator but not by transcription factors of the host cells. For the reasons discussed above, we believe that using a transactivator that is activated by certain antiprogestins for the co-control of RCCV replication represents a rational choice. If an RCCV of this type were administered to a subject, there would be essentially no possibility of run-away virus replication. Activation of the RCCV requires both triggers, i.e., an appropriate heat dose and an effective dose of an antiprogestin. Hence, even if the subject were exposed to a proteotoxic stress shortly after RCCV administration, e.g., experienced a severe fever, suffered from an exposure to a toxicant, engaged in particularly strenuous exercise, etc., virus replication would cease as soon as the antiprogestin concentration had fallen below the effective level. An accelerated decline of the antiprogestin concentration to an ineffective systemic level could be achieved by co-administering the drug with the RCCV rather than administering it orally. This should further minimize the possibility of inadvertent systemic replication of the virus. Replication in quiescently infected nerve cells should not be capable of occurring in the absence of antiprogestin (discussed in more detail below). Theoretically, some replication may take place in a stressed subject that ingests an effective dose of an antiprogestin (such as mifepristone and ulipristal). As long as such antiprogestins are essentially only used in single doses for emergency contraception, this possible complication would be de minimis.

### 5.1. Additional Thoughts on the Antiprogestin Co-Control of RCCVs

The appropriateness of employing an antiprogestin-activated transactivator in a RCCV-based vaccine may need to be re-evaluated in light of more recent developments. Pivotal clinical trials demonstrated that ulipristal is capable of reducing the size of uterine fibroids, resulting in marketing approvals of the ulipristal-containing drug Esmya (Gedeon Richter Plc, Budapest, Hungary) in Europe and Canada [43,44,45]. A typical treatment involves one or more treatment cycles of up to three months (5 mg/day of ulipristal taken orally). By 2018, multiple cases of serious liver injury had been reported that were ascribed to the long-term use of Esmya. Several patients required liver transplants as a consequence. An investigation by the European Medicines Agency (EMA) resulted in proposed measures to minimize the risk of severe liver injury as well as a requirement that a warning card be included in drug packages [46,47]. In several European countries, no new patients are to be prescribed Esmya [48]. The U.S. Food and Drug Administration (FDA) declined to approve the drug [49]. Bayer AG halted the further clinical development of its candidate drug for the treatment of uterine fibroids containing vilaprisan, a compound closely resembling ulipristal and mifepristone [50]. Meanwhile, Elagolix (AbbVie Inc, North Chicago, ILL, USA) and Relagulix (Myovant Sciences Inc, Brisbane, CA, USA), drugs containing a gonadotropin-releasing hormone antagonist as the active agent, are nearing approval in the U.S. and elsewhere for the same indication (Relagulix already having been approved in Japan) [51,52,53]. Therefore, it appears more likely than not that in a few years’ time, antiprogestin-containing drugs will no longer be prescribed for the treatment of uterine fibroids. It is important to note that no cases of severe liver impairment have ever been reported for ellaOne (HRA Pharma, Châtillon, France), the ulipristal-containing drug used in single doses for emergency contraception since 2009. Consequently, the safety of administering single doses of ulipristal remains unquestioned.

Should some of the above-mentioned antiprogestins continue to be used for the treatment of uterine fibroids or for other indications, further control features could be added in the current heat- and antiprogestin-activated RCCVs. For example, an additional replication-essential gene could be subjected to the control of an appropriate tissue-specific promoter to prevent virus replication in untargeted tissues, particularly in nerve tissue. Having this additional feature present should effectively insulate the RCCVs against any untoward effects of therapeutic uses of antiprogestins.

### 5.2. RCCVs Co-Controlled by an SMR Other Than an Antiprogestin

Transactivators have been described that respond to doxycycline, erythromycin, pristinamycin, cumate, phloretin, ecdysteroids, diacylhydrazines (nonsteroidal ecdysone agonists) such as RG-102277 (RSL-1), LG335 (synthetic retinoic X receptor (RXR) α ligand), 4-hydroxytamoxifen (antiestrogen), 4,4′-dihydroxybenzil (DHB), parabens such as ethyl-4-hydroxybenzoate or propyl-4-hydroxybenzoate (RXRβ ligands with low estrogenic activity), non-immunosuppressive rapalogs and FK506 derivatives (reviewed in Reference [54]).The only SMRs in the latter list that are approved for use in human drugs are the antibiotics (doxycycline, erythromycin, and pristinamycin) and the antiestrogen 4-hydroxytamoxifen. However, antibiotics are administered to patients suffering from bacterial infections, which can be associated with severe fever. 4-Hydroxytamoxifen is used in multi-dose regimens in a number of indications including hyperplasia of the breast, infertility, early and advanced estrogen receptor-positive breast cancer, gynecomastia, and peripheral precocious puberty. Among the non-approved substances, phloretin and ecdysteroids occur in natural products, have multiple pharmacological activities and are even offered in supplements. Parabens are present in many pharmaceutical and cosmetic products. Cumate (4-isopropylbenzoate) occurs in various plant species, in particular in cumin, which is present in certain foods as well as being used in spices and food supplements. Diacylhydrazines are active agents in commercially used insecticides. Transactivators responding to non-immunosuppressive derivatives of rapamycin and FK506 are also susceptible to activation by rapamycin and FK506, both of which are compounds used in human medicine. Transactivators that are activated by LG335 or DHB may be worth considering as possible substitutes for the antiprogestin-activated GLP65 transactivator present in the current RCCVs. LG335 was shown to activate a transactivator comprising a mutated RXRα [55]. The transactivator was not activated by the natural ligand 9-cis-retinoic acid (approved for the treatment of Kaposi’s sarcoma and chronic hand eczema), and the native receptor was not activated by LG335. Multiply mutated estrogen receptors were identified that have a greatly altered specificity favoring the non-steroidal compound DHB relative to the natural ligand 17β-estradiol > 10^7^-fold [56]. DHB-dependent transactivators comprising the ligand-binding domain of a multiply mutated estrogen receptor were developed [57]. These transactivators are not expected to be responsive to estrogen at concentrations that are attained in medicated individuals.

### 5.3. Reactivation from Quiescence in Infected Nerve Cells

Heat- and antiprogestin-co-controlled RCCVs should not be susceptible to reactivation from quiescence in infected nerve cells. The promoters of one or two replication-essential genes were replaced with minimal GAL4-responsive promoters in these RCCVs. To the best of our knowledge, there are no cellular or viral transcription factors that can mediate transcription from these promoters. A GAL4 promoter drives the gene for the major single-strand DNA-binding protein ICP8 in HSV-GS3. In HSV-GS7, the gene for capsid protein VP19c is under GAL4 promoter control. ICP8 and VP19c are essential for lytic virus replication and should be equally indispensable for replication of a reactivated virus. In HSV-GS1 and HSV-GS3, the genes for ICP4 are controlled by GAL4 promoters. To the extent that this is known, early viral regulators that include ICP4 play a critical role in the late stages of reactivation, in particular during reactivation induced by fever/hyperthermia [58,59,60,61]. It is noted that even if RCCV-infected nerve cells expressed a hitherto undiscovered transcription factor that is capable of transactivating a GAL4 promoter, no blisters or sores should appear, as these are a consequence of infection of cells on the body surface. The RCCVs are not capable of lytic replication without activation by heat and antiprogestin, as evidenced by our extensive in vitro and in vivo studies. Furthermore, immunization with an activated RCCV generates a strong, self-directed cellular immune response. This induced immunity may effectively suppress any possible reactivation of the RCCV in quiescently infected cells. Obviously, the strong expectation that quiescently present RCCVs will not reactivate in the absence of a deliberate activation treatment will need to be validated experimentally in appropriate models of latent infection.

### 5.4. How Could RCCV-Based Vaccination Be Practiced?

Finally, it may be worth reflecting about the practicality of RCCV-based vaccination and the manner in which it could be practiced. For the reasons discussed above, it may be advantageous to administer the antiprogestin directly to the site of vaccine inoculation rather than systemically. Hence, an appropriate antiprogestin formulation may be included in the vaccine composition. In our experiments, heat treatment to the inoculation site was applied several hours after virus administration. If this interval were respected, two visits to a physician’s office might be required, one for vaccine administration and a second for the heat treatment. However, a second visit with the physician could be avoided by providing inoculated subjects with a simple heating device such as a small pad that is heated by crystallization of a supercooled, nontoxic salt solution (as described in more detail previously [31]) as well as instructions for when and how to self-apply the device. Alternatively, it may be possible to more closely space vaccine administration and heat treatment, making it feasible to complete the entire vaccination procedure during a single visit. In this case, it would be important to minimize heat treatment duration. A dedicated high-intensity focused ultrasound or IR-based device may be developed that is capable of reproducibly delivering an activating heat pulse to the site of vaccine administration.
